# The EGFR Signaling Modulates in Mesenchymal Stem Cells the Expression of miRNAs Involved in the Interaction with Breast Cancer Cells

**DOI:** 10.3390/cancers14071851

**Published:** 2022-04-06

**Authors:** Marianna Gallo, Marianeve Carotenuto, Daniela Frezzetti, Rosa Camerlingo, Cristin Roma, Francesca Bergantino, Nicola Normanno, Antonella De Luca

**Affiliations:** Cell Biology and Biotherapy Unit, Istituto Nazionale Tumori-IRCCS-Fondazione G. Pascale, 80131 Naples, Italy; marianna.gallo@istitutotumori.na.it (M.G.); mn.carotenuto@gmail.com (M.C.); d.frezzetti@istitutotumori.na.it (D.F.); r.camerlingo@istitutotumori.na.it (R.C.); c.roma@istitutotumori.na.it (C.R.); f.bergantino@istitutotumori.na.it (F.B.); n.normanno@istitutotumori.na.it (N.N.)

**Keywords:** MSCs, EGFR, miRNAs, breast cancer cells, miR-23c

## Abstract

**Simple Summary:**

The epidermal growth factor receptor (EGFR) plays a central role in the tumor microenvironment, through the activation of paracrine and autocrine circuits that promote cancer development and progression. Our study for the first time provides evidence that the EGFR system induces in mesenchymal stem cells (MSCs) significant changes in the expression of a wide number of miRNAs, including miR-23c, that might be involved in the cross-talk with breast cancer cells. We also propose a novel mechanism of action of miR-23c in basal/claudin-low breast cancer cell lines. These results might help to increase our knowledge on the mechanisms of breast cancer progression mediated by the EGFR in the tumor microenvironment.

**Abstract:**

We previously demonstrated that the epidermal growth factor receptor (EGFR) modulates in mesenchymal stem cells (MSCs) the expression of a number of genes coding for secreted proteins that promote breast cancer progression. However, the role of the EGFR in modulating in MSCs the expression of miRNAs potentially involved in the progression of breast cancer remains largely unexplored. Following small RNA-sequencing, we identified 36 miRNAs differentially expressed between MSCs untreated or treated with the EGFR ligand transforming growth factor α (TGFα), with a fold change (FC) < 0.56 or FC ≥ 1.90 (CI, 95%). KEGG analysis revealed a significant enrichment in signaling pathways involved in cancer development and progression. EGFR activation in MSCs downregulated the expression of different miRNAs, including miR-23c. EGFR signaling also reduced the secretion of miR-23c in conditioned medium from MSCs. Functional assays demonstrated that miR-23c acts as tumor suppressor in basal/claudin-low MDA-MB-231 and MDA-MB-468 cells, through the repression of IL-6R. MiR-23c downregulation promoted cell proliferation, migration and invasion of these breast cancer cell lines. Collectively, our data suggested that the EGFR signaling regulates in MSCs the expression of miRNAs that might be involved in breast cancer progression, providing novel information on the mechanisms that regulate the MSC-tumor cell cross-talk.

## 1. Introduction

The epidermal growth factor receptor (EGFR) plays an important role in human tumors, by regulating cancer cell proliferation and survival [1]. In the tumor microenvironment, the EGFR system activates paracrine and autocrine circuits that promote tumor growth and progression [2]. Among cells in the tumor microenvironment, bone-marrow-derived mesenchymal stem cells (MSCs), adult multipotent stromal cells with self-renewal and differentiation ability, were found to express a functional EGFR [3,4]. Activation of the EGFR in MSCs stimulates the production of factors that promote angiogenesis and cell migration in different tumor types, including breast cancer [4,5,6]. The EGFR is also able to modulate in MSCs a wide array of genes coding for secreted proteins that may enhance tumor progression [7]. However, the role of the EGFR in modulating microRNAs (miRNAs) in MSCs remains largely unexplored.

MiRNAs are short single-stranded noncoding RNA molecules of 19–25 nucleotides that play a central role in the post-transcriptional regulation of gene expression, by binding to complementary target messenger RNAs (mRNAs) and leading to translational inhibition or mRNA degradation [8]. MiRNAs are frequently deregulated in human cancers [8]. In breast cancer, dysregulated miRNAs influence proliferation, survival and the invasive capacity of cancer cells through the overexpression or silencing of target genes [9,10,11].

Different studies demonstrated that miRNAs are involved in the interaction between cells of the tumor microenvironment and breast cancer cells via paracrine mechanisms. In this regard, MSC-upregulated miR-199a in breast cancer cells promoted cancer stem cell propagation and metastasis through the repression of the FOXP2 gene [11]. In addition, miR-148b-3p overexpressing exosomes from umbilical cord MSCs suppressed breast cancer cell proliferation, invasion and migration through the downregulation of the TRIM9 gene [12]. Moreover, MSC-derived miR-16 was involved in the downregulation of VEGF expression in breast cancer cells with a consequent inhibition of angiogenesis [13].

Starting from this evidence, we hypothesized that the EGFR was able to modulate in MSCs a set of miRNAs potentially involved in breast cancer progression. To address this hypothesis, we analyzed the expression profile of miRNAs in MSCs stimulated with the EGFR ligand TGFα, using a small RNA-sequencing approach. Among miRNAs modulated by EGFR activation in MSCs, we found miR-23c, whose biological activity in breast cancer cells has not been fully explored.

## 2. Materials and Methods

### 2.1. Cell Cultures

Bone-marrow-derived MSCs were purchased from Lonza (Verviers, Belgium) and maintained in culture as previously described [4]. The human breast cancer cell lines MDA-MB-231, MDA-MB-468, MCF-7, SKBR3, T47D and the human mammary epithelial cells MCF-10A were purchased from the American Type Culture Collection (ATCC, Manassas, VA, USA) and cultured as previously described [5,14]. All cell lines were maintained in a 5% CO_2_-humidified incubator at 37 °C.

### 2.2. Small RNA Isolation and Sequencing

Total RNA was extracted from serum-starved MSCs following treatment with 10 ng/mL recombinant human TGFα (PeproTech, Rocky Hill, NJ, USA) for 1 h, using the TRIzol Reagent (Invitrogen, Milan, Italy). The small RNA fraction was enriched using the PureLink miRNA Isolation Kit (Thermo Fisher Scientific, Milan, Italy). The quantity and quality of enriched samples were determined by the Agilent 2100 Bioanalyzer (Agilent Technologies, Milan, Italy). Then, samples were subjected to hybridization and ligation to SOLiD adaptor mix, using the SOLiD Small RNA library preparation protocol with the SOLiD Total RNA-Seq kit (Thermo Fisher Scientific). Libraries of cDNA were generated by reverse transcription and purified using the MinElute PCR Purification kit (Qiagen, Milan, Italy). Following size selection of cDNA, amplification was performed using SOLiD 5′PCR primers and barcoded SOLiD 3′PCR primers (Thermo Fisher Scientific). Amplified cDNA was purified using the PureLink PCR Micro Kit and quantified with the Qubit fluorometer (Thermo Fisher Scientific). Barcoded cDNA libraries were captured onto the surface of beads, amplified by emulsion PCR and enriched using the SOLiD EZ Beads System (Thermo Fisher Scientific). Beads were deposited onto glass slides and sequenced on the SOLiD 5500xl platform (Thermo Fisher Scientific) using the fragment protocol (35 bp).

### 2.3. Small RNA Data Analysis

Small RNA analysis was performed using the LifeScope Genomic Analysis Software small RNA analysis module (Thermo Fisher Scientific). Reads generated from irrelevant sources, including tRNAs and rRNAs, were eliminated through preliminary filtering. Sequencing reads were aligned to the Homo Sapiens genome reference GRCh38/hg38 and the dataset of mature sequences and precursors miRBase v21 was used to identify validated miRNAs (http://www.mirbase.org/, last accessed on 10 June 2020). Read counts were normalized using the TMM (Trimmed Mean of M-values) normalization and differential expression analysis was performed [15]. Only miRNAs with read counts greater than 150 in at least one sample and scored above the threshold score, set at 95% confidence, were withheld and considered differentially expressed between untreated and TGFα-treated MSCs.

### 2.4. MiRNA Target Prediction and Pathways Enrichment Analysis

Target genes of the differentially expressed miRNAs were identified using the miRWalk database v2.0 [16]. Predicted miRNA target genes were analyzed for enrichment in KEGG (Kyoto Encyclopedia of Genes and Genomes) pathways using the DAVID (Database for Annotation, Visualization and Integrated Discovery, https://david.ncifcrf.gov, last accessed on 20 November 2020) database.

### 2.5. Real-Time PCR

Total RNA was extracted using the TRIzol Reagent according to the manufacturer’s instruction. For miRNA analysis, total RNA was reverse transcribed with Taqman microRNA Reverse Transcription Kit and PCR was performed using the 7900 HT ABI PRISM and the TaqMan Universal PCR Master Mix II no UNG (Thermo Fisher Scientific). For mRNA quantization, RNA was reverse transcribed with the Super Script II Reverse Transcriptase and amplified with Power Syber Green PCR Master Mix (Thermo Fisher Scientific). Primer sequences will be supplied upon request. Relative quantification of transcripts was performed using the 2^−ΔΔCt^ method.

### 2.6. Collection of Conditioned Media

Conditioned media were collected from untreated and TGFα-treated MSCs cultured for 8 days in serum-free medium. Circulating cell-free miRNAs were extracted from conditioned media using the QIAamp Circulating Nucleic Acid kit (Qiagen), according to manufacturer’s instructions.

### 2.7. Cell Transfection with microRNA Mimics or Inhibitors

MiR-23c or miR-379-3p mimics (accession no. MIMAT0018000; accession no. MIMAT0004690) and miR-23c or miR-379-3p inhibitors (Anti-miR, accession no. MIMAT0018000 and accession no. MIMAT0004690) were purchased from Thermo Fisher Scientific. Random sequences of miRNA mimic or inhibitor were used as non-targeting negative controls (NTCs).

MDA-MB-468 and MDA-MB-231 cells (2 × 10^4^ cells/well) were seeded into 6-well plates and allowed to adhere overnight until they reached 50% confluence. The transfection of miRNA mimics (60 pmol/mL) or inhibitors (200 pmol/mL) and their relative NTCs of breast cancer cells was performed using the Lipofectamine RNAiMax Transfection Reagent (Invitrogen). After 72 h, cells transfected with miR-23c or miR-379-3p mimics or inhibitors and their respective NTCs were harvested for subsequent experiments.

### 2.8. Cell Proliferation, Migration and Invasion Assays

For cell proliferation assays, transfected cells were seeded into 96-well plates (6 × 10^3^ cells/well) in serum-containing medium. Cell proliferation was measured at different time points, using the tetrazolium-based (MTT) colorimetric assay as previously described [14].

Cell migration was determined using the Colorimetric Cell Migration Assay (Chemicon/Millipore, Milan, Italy). Briefly, transfected cells were seeded in the upper wells (3 × 10^4^ cells) in 0.5% FBS-containing medium and allowed to migrate for 16 h through the inserts in the lower compartment filled with 10% FBS. Cells migrated across the membrane were stained with a crystal violet stain solution. The absorbance was read at 540 nm in each well.

The Cell Invasion Assay Kit (Millipore) was used for measuring cell invasion, as previously described [17]. Transfected cells were seeded in upper chambers (4 × 10^4^ cells) and allowed to invade for 45 h through a matrigel-coated membrane. Medium supplemented with 10% FBS was added as chemoattractant in the lower compartment.

### 2.9. Flow Cytometry Analysis

Transfected cells were detached by incubation with 0.02% EDTA in PBS, centrifuged and washed in PBS containing 0.5% BSA and 0.1% sodium azide. Cells were incubated for 1 h at 4 °C with the mouse anti-human IL-6R PE (Miltenyi Biotec, Bologna, Italy). Cytofluorimetric analysis was performed with the BD FACS ARIA III Cell Sorter and the DiVa 8.0 software (Becton Dickinson, Mountain View, CA, USA).

### 2.10. Western Blot Analysis

Whole protein extracts were prepared and analyzed by Western blotting, according to a standard procedure. The following antibodies were used: anti-IL-6R (Abcam, Cambridge, UK); anti-phospho STAT3 (Tyr705, clone 3E2) and anti-STAT3 (Cell Signaling Technology, Danvers, MA, USA); anti-α-tubulin clone DM1A (Sigma-Aldrich, Milan, Italy). Densitometric analysis was performed using the ImageJ software. The original Western blotting images are shown in Figure S3.

### 2.11. Luciferase Reporter Assay

The 3′-UTR of IL-6R mRNA containing putative miR-23c binding sites was PCR-amplified from human genomic DNA with the AmpliTaq Gold DNA Polymerase (Thermo Fisher). The 3′-UTR was cloned into the pmirGLO Dual-Luciferase miRNA Target Expression vector (Promega, Madison, WI, USA) to obtain the WT IL-6R UTR-pmirGLO plasmid. The complementary miR-23c binding site was mutated using the Quick-Change Lightning Site-Directed Mutagenesis Kit (Agilent Technologies) to obtain the MUT IL-6R UTR-pmirGLO vector. For the luciferase reporter assay, MDA-MB-468 cells were co-transfected with WT/MUT IL-6R plasmids and miR-23c mimic or NTC. After 48 h, luciferase activity was determined using the Dual-Glo Luciferase Assay System (Promega) according to manufacturer’s instructions. All transfections were performed in triplicate and were normalized to Renilla luciferase activity.

### 2.12. Statistical Analysis

Statistical significance was determined using the two-tailed Student’s *t*-test and *p* values ≤ 0.05 were considered statistically significant.

## 3. Results

### 3.1. MiRNA Expression Profiling of MSCs following Stimulation with TGFα

The expression profile of miRNAs in MSCs stimulated with TGFα was analyzed using a small RNA-sequencing approach. Sequencing of small RNA libraries with the SOLiD 5500xl platform yielded 22,022,204 and 25,067,668 sequencing reads for untreated- and TGFα-treated MSCs, respectively. The Quality Value for all the obtained reads resulted at least ≥10 for the first 20 bases. The mapping of the sequence reads to the reference genome GRCh38/hg38 evidenced a percentage of total miRNA mapping reads of 64% for MSCs and 52% for TGFα-treated MSCs corresponding to 14,142,107 and 13,023,348 reads, respectively. Among them, 11,053,070 (78.2%) reads for untreated MSCs and 10,195,770 (78.3%) reads for TGFα-treated MSCs were considered as annotated reads, perfectly aligned with known mature miRNAs (miRBase v21.0). Using this tool, we identified 1425 mature miRNAs with at least one count value > 0 that were differentially expressed between untreated and TGFα-treated MSCs (Supplementary Table S1).

**Figure 1:**
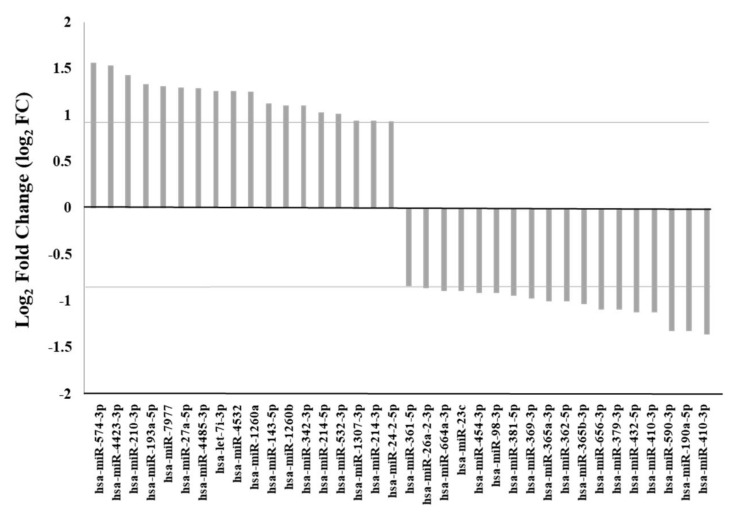
Differentially expressed microRNAs (miRNAs) between MSCs untreated and treated with TGFα. Eighteen miRNAs resulted upregulated in MSCs following treatment with TGFα with a log2 fold change (FC) ≥ 0.93 and 18 downregulated with a log2FC < −0.83 (Confidence Interval, CI, 95%).

Count normalization, performed using the TMM tool, showed a high fold change concordance between 3p and 5p, arising from the same miRNA. Applying a threshold of sequence counts ≥ 150 in at least one sample, chosen to reduce miRNAs with very low levels of expression, we identified 36 differentially expressed miRNAs with a log2 fold change (FC) ≥ 0.93 or <−0.83 (Confidence Interval, CI, 95%). In particular, 18 miRNAs resulted upregulated and 18 miRNAs downregulated by EGFR activation in MSCs (Figure 1). We observed that the majority of these differentially expressed miRNAs were involved in cancer development and progression. Interestingly, 20 out 36 of the differentially expressed miRNAs, modulated by the EGFR in MSCs, were included in the list of 466 miRNAs associated with tumorigenesis of invasive breast cancer in the OncomiR database (www.oncomir.org, last accessed on 15 September 2021). Target genes of the 36 differentially expressed miRNAs were identified using the miRWalk v2.0 database. Targets predicted by at least four of the five miRNA target prediction programs selected (miRWalk, miRanda, miRDB, PITA and TargetScan) with a *p* value < 0.05 were considered. Collectively, we found that the 36 differentially expressed miRNAs had 9532 target genes. To identify biochemical pathways in which target genes of the differentially expressed miRNAs were involved, we performed a KEGG (Kyoto Encyclopedia of Genes and Genomes) pathway enrichment analysis using the DAVID database. Following treatment with TGFα, we observed in MSCs a significant enrichment in several pathways, such as TGFβ signaling, focal adhesion, Rap1 signaling, Hippo signaling, mTOR and RAS signaling, indicating that the EGFR system modulated in MSCs miRNAs potentially involved in cancer development and progression.

### 3.2. Analysis of miRNAs in Conditioned Media from MSCs

**Figure 2:**
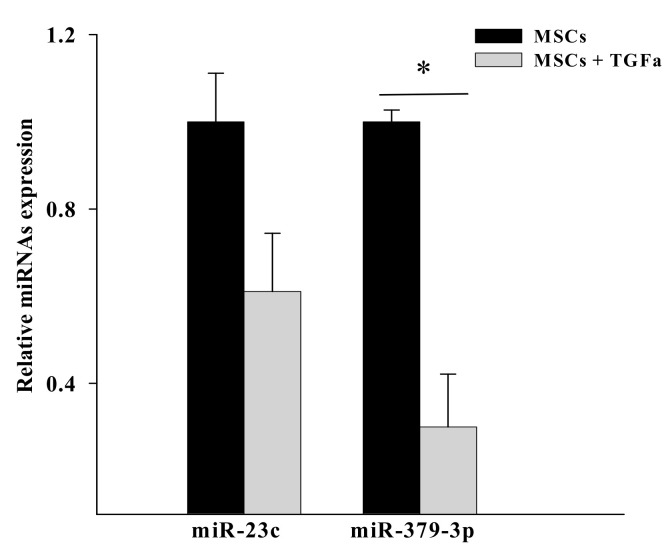
Expression levels of miR-23c and miR-379-3p in conditioned media from untreated and TGFα-treated MSCs. The levels of the expression of miR-23c and miR-379-3p were quantified by Real-time PCR. Conditioned medium from untreated MSCs was used as calibrator. (* *p* ≤ 0.05 for comparison between untreated versus TGFα-treated MSCs, two-tailed Student’s *t*-test).

To assess whether EGFR-regulated miRNAs in MSCs were involved in the cross-talk with breast cancer cells, we analyzed the levels of expression of the differentially expressed miRNAs in conditioned media from untreated and TGFα-treated MSCs, particularly focusing on miRNAs that we found to be associated with breast cancer in the OncomiR database, including miR-23c and miR-379-3p. We found that MSCs were able to secrete miR-23c and miR-379-3p in the conditioned media. Moreover, in agreement with sequencing data, we found in conditioned media from TGFα-treated MSCs lower levels of expression of miR-23c and miR-379-3p, as compared with untreated cells (Figure 2).

### 3.3. Functional Analysis of miR-23c and miR-379-3p

**Figure 3:**
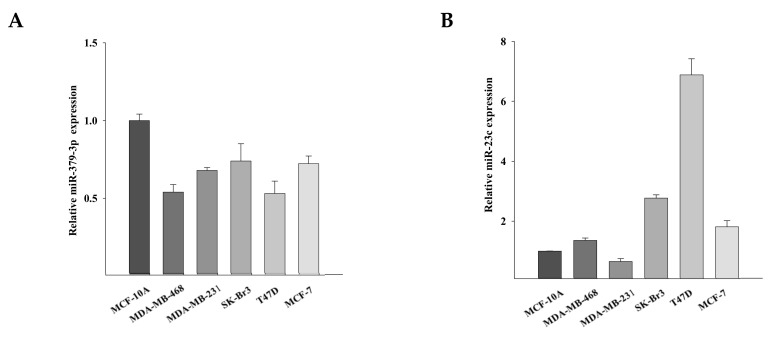
Levels of expression of miR-379-3p and miR-23c in breast cancer subtypes. (**A**,**B**) Real-time PCR analysis of miR-379-3p (**A**) and miR-23c (**B**) expression in luminal (MCF-7 and T47D), HER2 positive (SKBr3), basal (MDA-MB-468) and claudin-low (MDA-MB-231) breast cancer cell lines compared with the human breast epithelial cell line MCF-10A.

Based on the observation that the levels of expression of miR-23c and miR-379-3p were reduced in conditioned media from TGFα-stimulated MSCs, we investigated the effects of the downregulation of these miRNAs in breast cancer cells. We first analyzed the levels of expression of miR-379-3p and miR-23c in cell lines belonging to different breast cancer subtypes, such as luminal (MCF-7 and T47D), HER2 positive (SKBr3), basal (MDA-MB-468) and claudin-low (MDA-MB-231) cell lines. We found that miR-379-3p was downregulated in all analyzed breast cancer cell lines, compared with the human breast epithelial cell line MCF-10A (Figure 3A). MiR-23c was upregulated in MCF-7, T47D and SKBr3 breast cancer cells, as compared with MCF-10A cells. Interestingly, the levels of expression of miR-23c in the MDA-MB-468 cell line were comparable to MCF10A cells, whereas in MDA-MB-231 cells were lower as compared to that observed in less aggressive breast cancer cell lines and in epithelial cells (Figure 3B).

**Figure 4:**
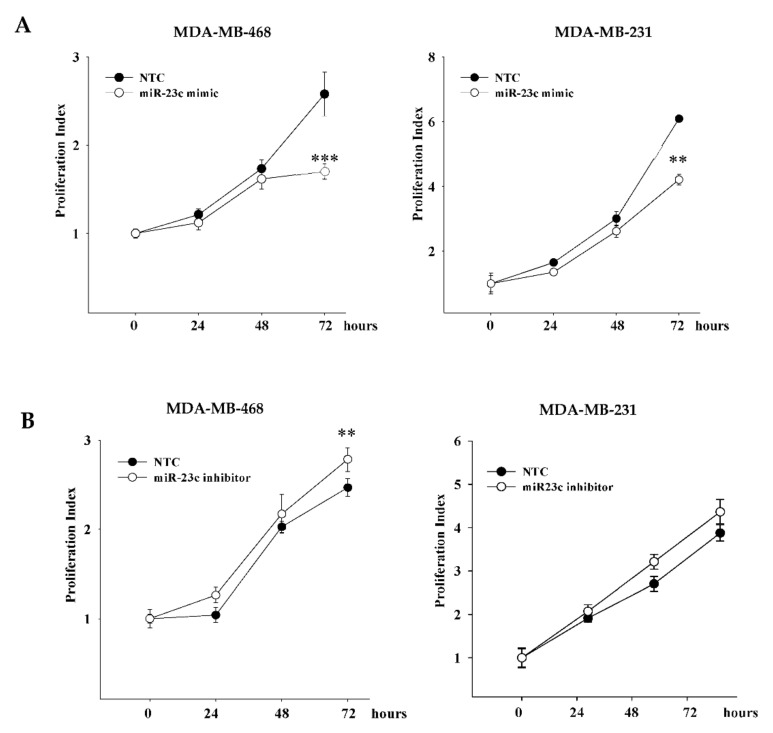
Effects of miR-23c on breast cancer cell proliferation. The proliferation rates of MDA-MB-468 and MDA-MB-231 cells transfected with miR-23c mimic (**A**) or miR-23c inhibitor (**B**) and their respective non-targeting controls (NTCs) were measured for 72 h after transfection. Proliferation index was determined measuring OD at the indicated time points and calculating the ratio compared to zero time point. (** *p* ≤ 0.005 and *** *p* ≤ 0.001 for comparison between untreated versus TGFα-treated MSCs, two-tailed Student’s *t*-test).

As the tumor suppressive role of miR-379-3p in breast cancer cells has been previously demonstrated [18,19], we sought to focus our attention on miR-23c, whose biological function in breast cancer cells has not been explored. We transiently transfected MDA-MB-468 and MDA-MB-231 cells that had endogenous levels of expression of miR-23c lower than the other breast cancer cell lines, with miR-23c mimic or inhibitor. We found that the overexpression of miR-23c inhibited the proliferation of MDA-MB-468 and MDA-MB-231 cells (Figure 4A), compared with cells transfected with the NTC, whereas the knockdown of miR-23c slightly increased cell proliferation (Figure 4B).

**Figure 5:**
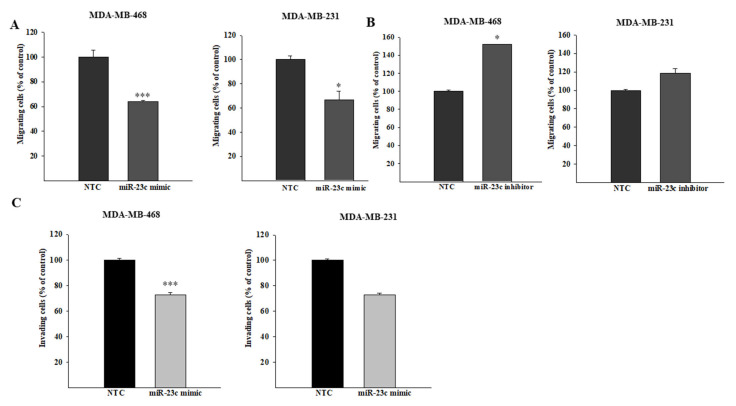
MiR-23c regulates migration and invasion of breast cancer cells. MDA-MB-468 and MDA-MB-231 cells were transfected for 72 h with miR-23c mimic (**A**) or inhibitor (**B**) and their respective non-targeting controls (NTCs) and then allowed to migrate through an insert toward serum-containing medium for 16 h (* *p* ≤ 0.05 and *** *p* ≤ 0.001; Student’s *t*-test). (**C**) The invasive ability of MDA-MB-468 and MDA-MB-231 cell lines to transfect miR-23c mimic or NTC was determined using a Boyden chamber-based colorimetric assay (*** *p* ≤ 0.001; Student’s *t*-test).

A significant reduction of the migratory ability was observed in both cell lines transfected with the miR-23c mimic (Figure 5A), whereas the inhibition of miR-23c induced an increase of cell migration in MDA-MB-468 and MDA-MB-231 cells (Figure 5B). Interestingly, a significant reduction of cell invasion was observed in both breast cancer cell lines transfected with the miR-23c mimic, as compared to control cells (Figure 5C).

To address whether miR-379-3p acts as tumor suppressor in our experimental model, we performed functional experiments in MDA-MB-468 and MDA-MB-231 cells transfected with miR-379-3p mimic or inhibitor. In agreement with previous data, the overexpression of miR-379-3p reduced breast cancer cell proliferation, migration and invasion (Supplementary Figures S1 and S2).

Collectively, our data supported the hypothesis that miR-23c had a tumor suppressive role in MDA-MB-468 and MDA-MB-231 cells, inhibiting cell proliferation, migration and invasion, and confirmed that miR-379-3p acts as tumor suppressor in these cells.

### 3.4. IL-6R Is a Target of miR-23c

**Figure 6:**
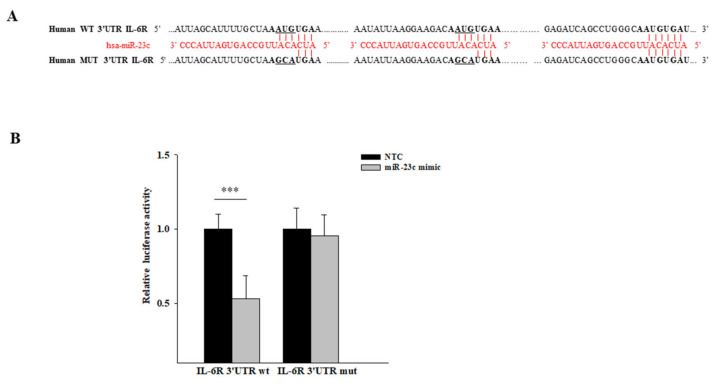
IL-6R is a target of miR-23c. (**A**) The putative 3′ binding sites of miR-23c in the IL-6R mRNA are displayed. Mutated bases in the 3′-UTR are underlined. (**B**) A dual luciferase reporter assay was performed to validate the interaction between miR-23c and IL-6R in MDA-MB-468 transfected with miR-23c mimic or non-targeting control (NTC) (*** *p* ≤ 0.001; Student’s *t*-test).

To identify the molecular mechanisms through which miR-23c regulates the proliferation, migration and invasion of MDA-MB-468 and MDA-MB-231 cell lines, we analyzed its potential target genes using the MiRWalk database. MiRWalk recognized 614 target genes of miR-23c, with a consensus of at least four miRNA target prediction programs. Among these, we identified as the candidate miR-23c target Interleukin 6 Receptor (IL-6R), whose ligand, IL-6, has been shown to play a pivotal role in breast cancer growth and metastasis. In addition, the increased expression of IL-6R correlated with disease progression and poor patient outcome in breast cancer [20]. To confirm that IL-6R is a target of miR-23c, we cloned the 3′-UTR of IL-6R (WT) or the corresponding mutant construct (MUT), containing a mutation in two of the three putative miR-23c binding sites, in a luciferase reporter plasmid (Figure 6A). Then, we performed a luciferase assay in MDA-MB-468 cells. We observed that the miR-23c mimic significantly reduced the luciferase activity in MDA-MB-468 cells transfected with the plasmid encoding the IL-6R 3′-UTR WT but not in cells transfected with the mutant plasmid (Figure 6B). These results demonstrated that IL-6R was a direct target of miR-23c.

**Figure 7:**
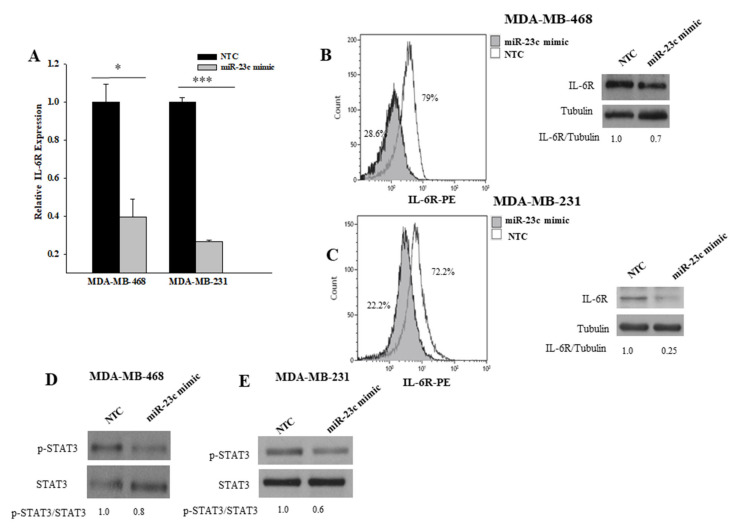
Effects of miR-23c on IL-6R expression and downstream signaling proteins. (**A**) The transcript of IL-6R in MDA-MB-231 and MDA-MB-468 cells, transfected with the miR-23c mimic or the non-targeting control (NTC), was measured by Real-time PCR (* *p* ≤ 0.05 and *** *p* ≤ 0.001; Students *t*-test). (**B**) Cytofluorimetric and Western blot analysis of IL-6R protein in MDA-MB-468 cells transfected with the miR-23c mimic or the NTC. Blot was normalized with the α-tubulin antibody. Quantification was performed using densitometric analysis. Densitometric value ratios for IL-6R/tubulin are shown. (**C**) Cytofluorimetric and Western blot analysis of IL-6R protein in MDA-MB-231 cells transfected with the miR-23c mimic or the NTC. Densitometric value ratios for IL-6R/tubulin are shown. Western blot analysis of activated form of STAT3 in MDA-MB-468 (**D**) and MDA-MB-231 cells (**E**) transfected with the miR-23c mimic or the NTC. Blots were normalized with the total STAT3 antibody. Densitometric value ratios for p-STAT3/total STAT3 are shown for each panel (**D**,**E**).

To evaluate whether miR-23c was able to negatively regulate the expression of endogenous IL-6R in breast cancer cell lines, we analyzed the levels of expression of IL-6R transcript by Real-time PCR and protein by cytofluorimetry and Western blotting in MDA-MB-468 and MDA-MB-231 cells transfected with the miR-23c mimic (Figure 7A–C). We observed that the overexpression of miR-23c significantly reduced both the IL-6R transcript and protein in both cell lines. (Figure 7A–C).

Finally, we analyzed the effects of the overexpression of miR-23c on STAT3, a signaling protein activated by IL-6R. The overexpression of miR-23c resulted in a significant decrease of STAT3 phosphorylation as compared with cells transfected with the NTC in MDA-MD-231 cells, whereas a non-significant reduction of phospho-STAT3 was observed in MDA-MB-468 cells, thus suggesting that miR-23c inhibited STAT3 signaling via downregulation of IL-6R (Figure 7D,E).

## 4. Discussion

Different studies demonstrated that the EGFR modulates in breast cancer cells the expression of miRNAs involved in cancer progression. In this regard, EGF-induced miR-15b was shown to stimulate the migration of breast cancer cells, through the suppression of the MTSS1 gene [21]. Furthermore, miR-338-3p, modulated by the EGF in MCF-7 cells, promoted breast cancer cell growth and epithelial-to-mesenchymal transition, migration and invasion [22]. However, the paracrine effects on breast cancer cells of EGFR-regulated miRNAs in MSCs have not been fully explored.

In this study, we demonstrated for the first time that EGFR activation regulates in MSCs the expression of a wide number of miRNAs potentially involved in the cross-talk with breast cancer cells. In particular, we found that the EGFR modulates in MSCs the expression of miRNAs involved in breast cancer tumorigenesis, as indicated in the OncoMiR database, progression and response to therapeutic regimens, such as miR-574-3p, miR-342-3p and miR-210-3p [23,24,25]. Bioinformatics analysis also revealed an enrichment of several signaling pathways that play an important role in cancer cell growth, differentiation, apoptosis and motility. These results strongly support the hypothesis that the EGFR might promote breast cancer progression through the regulation of miRNAs in cells of the tumor microenvironment.

Among miRNAs downregulated by the EGFR in MSCs, we found miR-23c. Few studies analyzed the role of miR-23c in the development and progression of cancer. In this regard, a recent study demonstrated that miR-23c acts as tumor suppressor in hepatocarcinoma cell lines, by inhibiting cell proliferation and inducing apoptosis through the regulation of the ERBB2IP gene [26]. In addition, miR-23c secreted from prostate cancer cells expressing regucalcin, a gene involved in tumor dormancy, was found to suppress the angiogenesis in human umbilical vein endothelial cells [27].

To our knowledge, the functional role of miR-23c in breast cancer cells has not been explored yet. Although TCGA data in the OncomiR database indicated that miR-23c was upregulated in breast cancer, as compared with its normal counterpart, we found that basal/claudin-low breast cancer cells had lower levels of expression of miR-23c, as compared with breast cancer cell lines belonging to less aggressive breast cancer subtypes. Based on these findings, we hypothesized that miR-23c might act as tumor suppressor in MDA-MB-468 and MDA-MB-231 cells, and functional experiments confirmed this hypothesis. In agreement with our observation, a recent study demonstrated that miR-23c was differentially expressed between triple negative (TNBC) and quadruple negative breast cancer (QNBC) [28]. Interestingly, in the analysis on the relationship between the expression levels of miR-23c and the clinicopathological characteristics of QNBC patients, high levels of expression of miR-23c were associated with the absence of metastases [28]. Therefore, it might be possible that miR-23c acts as tumor suppressor in TNBC cell lines and that EGFR activation in MSCs contributes to breast cancer progression through the downregulation of such miRNA in the microenvironment. In addition, as MDA-MB-468 and MDA-MB-231 cells express EGF-related ligands [29,30], we can hypothesize that breast cancer cells, through the secretion of TGFα, educate MSCs to promote breast cancer progression by repressing miRNAs that act as tumor suppressors, such as miR-23c. The fact that the same miRNAs might act as tumor suppressors or as oncogenes might depend on the context. In this regard, it is possible that the same miRNA has opposing oncogenic or tumor suppressive functions in various tumors or within a single cancer type [31]. This phenomenon can be explained because a single miRNA triggers a variety of targets that in turn activate different signaling pathways. Indeed, a recent study demonstrated that miR-31 had different functional roles across lung cancer subtypes by activating different signaling pathways [32].

Following bioinformatics and functional experiments, we found that IL-6R is a target of miR-23c. IL-6R is involved in the IL-6/IL-6R/STAT3 signaling pathway, whose role in breast cancer progression has been extensively demonstrated [20,33]. In addition, STAT3 signaling contributes to cell proliferation, survival, migration and invasion of TNBC [34,35]. In this regard, it has been shown that IL-6 stimulated the proliferation and migration of breast cancer cells [5,36,37]. In addition, IL-6 promoted breast cancer progression through the induction of the epithelial to mesenchymal transition [38]. Our data suggest that miR-23c may interfere with IL-6R/STAT3 signaling in breast cancer cell lines and that the upregulation of miR-23c in these cells reduces cell proliferation and migration by inhibiting IL-6R/STAT3 signaling.

The identification of miRNAs in the tumor microenvironment might have potential clinical implications. In this regard, liquid biopsy is a minimally invasive approach that relies on the analysis of cancer biomarkers in body fluids. Recent evidence suggested that circulating miRNAs are particularly stable in biological fluids, including plasma and serum, and may represent potential biomarkers using liquid biopsy approaches [39]. Detection of circulating miRNAs might have prognostic implications. In this regard, circulating miRNA signatures have been associated with the outcome of TNBC patients [40,41]. In addition, some studies explored the potential of circulating miRNAs for monitoring the response to treatment using liquid biopsy [40]. In this context, the analysis of the levels of expression of miR-23c might be potentially useful for providing prognostic or predictive information in TNBC patients.

## 5. Conclusions

In conclusion, our data demonstrated that the EGFR signaling regulates in MSCs a wide number of miRNAs that might be involved in breast cancer progression. Among miRNAs potentially involved in the cross-talk between EGFR-stimulated MSCs and breast cancer cells, we identified miR-23c and suggested a role of this miRNA as a tumor suppressor in MDA-MB-468 and MDA-MB-231 breast cancer cells, through the inhibition of IL-6R. Our results finally suggested that the EGFR downregulated in MSCs the expression of miR-23c, thus promoting cancer progression in highly aggressive breast cancer cells.

Collectively, our findings provide novel information on the mechanisms mediated by the EGFR in the tumor microenvironment that might be involved in breast cancer progression.

## Data Availability

The data presented in this study are available in the article and Supplementary Material.

## Supplementary Materials

The following supporting information can be downloaded at: https://www.mdpi.com/article/10.3390/cancers14071851/s1, Table S1. List of MiRNAs differentially expressed between untreated and TGFα-treated MSCs; Figure S1. Effects of miR-379-3p on the proliferation of breast cancer cell lines. Figure S2. Effects of miR-379-3p on the ability of breast cancer cells to migrate and invade. Figure S3: Original immunoblotting images (DOI: 10.5281/zenodo.6381931).

## References

[1] Hynes N.E., Lane H.A. (2005). ERBB receptors and cancer: The complexity of targeted inhibitors. Nat. Rev. Cancer.

[2] De Luca A., Carotenuto A., Rachiglio A., Gallo M., Maiello M.R., Aldinucci D., Pinto A., Normanno N. (2008). The role of the EGFR signaling in tumor microenvironment. J. Cell. Physiol..

[3] Satomura K., Derubeis A.R., Fedarko N.S., Ibaraki-O’Connor K., Kuznetsov S.A., Rowe D.W., Young M.F., Gehron Robey P. (1998). Receptor tyrosine kinase expression in human bone marrow stromal cells. J. Cell. Physiol..

[4] De Luca A., Gallo M., Aldinucci D., Ribatti D., Lamura L., D’Alessio A., De Filippi R., Pinto A., Normanno N. (2011). Role of the EGFR ligand/receptor system in the secretion of angiogenic factors in mesenchymal stem cells. J. Cell. Physiol..

[5] De Luca A., Lamura L., Gallo M., Maffia V., Normanno N. (2012). Mesenchymal stem cell-derived interleukin-6 and vascular endothelial growth factor promote breast cancer cell migration. J. Cell. Biochem..

[6] Kerpedjieva S.S., Kim D.S., Barbeau D.J., Tamama K. (2012). EGFR ligands drive multipotential stromal cells to produce multiple growth factors and cytokines via early growth response-1. Stem. Cells Dev..

[7] De Luca A., Roma C., Gallo M., Fenizia F., Bergantino F., Frezzetti D., Costantini S., Normanno N. (2014). RNA-seq analysis reveals significant effects of EGFR signalling on the secretome of mesenchymal stem cells. Oncotarget.

[8] Esquela-Kerscher A., Slack F.J. (2006). Oncomirs—microRNAs with a role in cancer. Nat. Rev. Cancer.

[9] Weidle U.H., Dickopf S., Hintermair C., Kollmorgen G., Birzele F., Brinkmann U. (2018). The Role of micro RNAs in Breast Cancer Metastasis: Preclinical Validation and Potential Therapeutic Targets. Cancer Genom. Proteom..

[10] Ma L., Teruya-Feldstein J., Weinberg R.A. (2007). Tumour invasion and metastasis initiated by microRNA-10b in breast cancer. Nature.

[11] Tavazoie S.F., Alarcon C., Oskarsson T., Padua D., Wang Q., Bos P.D., Gerald W.L., Massague J. (2008). Endogenous human microRNAs that suppress breast cancer metastasis. Nature.

[12] Yuan L., Liu Y., Qu Y., Liu L., Li H. (2019). Exosomes Derived From MicroRNA-148b-3p-Overexpressing Human Umbilical Cord Mesenchymal Stem Cells Restrain Breast Cancer Progression. Front. Oncol..

[13] Lee J.K., Park S.R., Jung B.K., Jeon Y.K., Lee Y.S., Kim M.K., Kim Y.G., Jang J.Y., Kim C.W. (2013). Exosomes derived from mesenchymal stem cells suppress angiogenesis by down-regulating VEGF expression in breast cancer cells. PLoS ONE.

[14] Normanno N., De Luca A., Maiello M.R., Campiglio M., Napolitano M., Mancino M., Carotenuto A., Viglietto G., Menard S. (2006). The MEK/MAPK pathway is involved in the resistance of breast cancer cells to the EGFR tyrosine kinase inhibitor gefitinib. J. Cell. Physiol..

[15] Tam S., Tsao M.S., McPherson J.D. (2015). Optimization of miRNA-seq data preprocessing. Brief. Bioinform..

[16] Dweep H., Sticht C., Pandey P., Gretz N. (2011). miRWalk--database: Prediction of possible miRNA binding sites by “walking” the genes of three genomes. J. Biomed. Inform..

[17] De Luca A., Lamura L., Strizzi L., Roma C., D’Antonio A., Margaryan N., Pirozzi G., Hsu M.Y., Botti G., Mari E. (2011). Expression and functional role of CRIPTO-1 in cutaneous melanoma. Br. J. Cancer.

[18] O’Brien K.P., Khan S., Gilligan K.E., Zafar H., Lalor P., Glynn C., O’Flatharta C., Ingoldsby H., Dockery P., De Bhulbh A. (2018). Employing mesenchymal stem cells to support tumor-targeted delivery of extracellular vesicle (EV)-encapsulated microRNA-379. Oncogene.

[19] Khan S., Brougham C.L., Ryan J., Sahrudin A., O’Neill G., Wall D., Curran C., Newell J., Kerin M.J., Dwyer R.M. (2013). miR-379 regulates cyclin B1 expression and is decreased in breast cancer. PLoS ONE.

[20] To S.Q., Dmello R.S., Richards A.K., Ernst M., Chand A.L. (2022). STAT3 Signaling in Breast Cancer: Multicellular Actions and Therapeutic Potential. Cancers.

[21] Kedmi M., Ben-Chetrit N., Korner C., Mancini M., Ben-Moshe N.B., Lauriola M., Lavi S., Biagioni F., Carvalho S., Cohen-Dvashi H. (2015). EGF induces microRNAs that target suppressors of cell migration: miR-15b targets MTSS1 in breast cancer. Sci. Signal..

[22] Liang Y., Xu X., Wang T., Li Y., You W., Fu J., Liu Y., Jin S., Ji Q., Zhao W. (2017). The EGFR/miR-338-3p/EYA2 axis controls breast tumor growth and lung metastasis. Cell Death Dis..

[23] Ujihira T., Ikeda K., Suzuki T., Yamaga R., Sato W., Horie-Inoue K., Shigekawa T., Osaki A., Saeki T., Okamoto K. (2015). MicroRNA-574-3p, identified by microRNA library-based functional screening, modulates tamoxifen response in breast cancer. Sci. Rep..

[24] Pasculli B., Barbano R., Rendina M., Fontana A., Copetti M., Mazza T., Valori V.M., Morritti M., Maiello E., Graziano P. (2019). Hsa-miR-210-3p expression in breast cancer and its putative association with worse outcome in patients treated with Docetaxel. Sci. Rep..

[25] Yu S., Zhou Y., Niu L., Qiao Y., Yan Y. (2022). Mesenchymal stem cell-derived exosome mir-342-3p inhibits metastasis and chemo-resistance of breast cancer through regulating ID4. Genes Genom..

[26] Zhang L., Wang Y., Wang L., Yin G., Li W., Xian Y., Yang W., Liu Q. (2018). miR-23c suppresses tumor growth of human hepatocellular carcinoma by attenuating ERBB2IP. Biomed. Pharmacother..

[27] Sharma S., Pei X., Xing F., Wu S.Y., Wu K., Tyagi A., Zhao D., Deshpande R., Ruiz M.G., Singh R. (2021). Regucalcin promotes dormancy of prostate cancer. Oncogene.

[28] Bhattarai S., Sugita B.M., Bortoletto S.M., Fonseca A.S., Cavalli L.R., Aneja R. (2021). QNBC Is Associated with High Genomic Instability Characterized by Copy Number Alterations and miRNA Deregulation. Int. J. Mol. Sci..

[29] De Luca A., Casamassimi A., Selvam M.P., Losito S., Ciardiello F., Agrawal S., Salomon D.S., Normanno N. (1999). EGF-related peptides are involved in the proliferation and survival of MDA-MB-468 human breast carcinoma cells. Int. J. Cancer.

[30] Ignacio R.M.C., Gibbs C.R., Lee E.S., Son D.S. (2018). The TGFα-EGFR-Akt signaling axis plays a role in enhancing proinflammatory chemokines in triple-negative breast cancer cells. Oncotarget.

[31] Svoronos A.A., Engelman D.M., Slack F.J. (2016). OncomiR or Tumor Suppressor? The Duplicity of MicroRNAs in Cancer. Cancer Res..

[32] Davenport M.L., Echols J.B., Silva A.D., Anderson J.C., Owens P., Yates C., Wei Q., Harada S., Hurst D.R., Edmonds M.D. (2021). miR-31 Displays Subtype Specificity in Lung Cancer. Cancer Res..

[33] Johnson D.E., O’Keefe R.A., Grandis J.R. (2018). Targeting the IL-6/JAK/STAT3 signalling axis in cancer. Nat. Rev. Clin. Oncol..

[34] Qin J.J., Yan L., Zhang J., Zhang W.D. (2019). STAT3 as a potential therapeutic target in triple negative breast cancer: A systematic review. J. Exp. Clin. Cancer Res..

[35] Hartman Z.C., Poage G.M., den Hollander P., Tsimelzon A., Hill J., Panupinthu N., Zhang Y., Mazumdar A., Hilsenbeck S.G., Mills G.B. (2013). Growth of triple-negative breast cancer cells relies upon coordinate autocrine expression of the proinflammatory cytokines IL-6 and IL-8. Cancer Res..

[36] Badache A., Hynes N.E. (2001). Interleukin 6 inhibits proliferation and, in cooperation with an epidermal growth factor receptor autocrine loop, increases migration of T47D breast cancer cells. Cancer Res..

[37] Sasser A.K., Sullivan N.J., Studebaker A.W., Hendey L.F., Axel A.E., Hall B.M. (2007). Interleukin-6 is a potent growth factor for ER-alpha-positive human breast cancer. FASEB J..

[38] Sullivan N.J., Sasser A.K., Axel A.E., Vesuna F., Raman V., Ramirez N., Oberyszyn T.M., Hall B.M. (2009). Interleukin-6 induces an epithelial-mesenchymal transition phenotype in human breast cancer cells. Oncogene.

[39] Heitzer E., Haque I.S., Roberts C.E.S., Speicher M.R. (2019). Current and future perspectives of liquid biopsies in genomics-driven oncology. Nat. Rev. Genet..

[40] Seale K.N., Tkaczuk K.H.R. (2022). Circulating Biomarkers in Breast Cancer. Clin. Breast Cancer.

[41] Gahlawat A.W., Fahed L., Witte T., Schott S. (2022). Total circulating microRNA level as an independent prognostic marker for risk stratification in breast cancer. Br. J. Cancer.

