# Supplementary material for: The EGFR Signaling Modulates in Mesenchymal Stem Cells the Expression of miRNAs Involved in the Interaction with Breast Cancer Cells
Source: Cancers (Basel). 2022 Apr 6;14(7):1851. doi: 10.3390/cancers14071851 (PMC8997927; doi:10.3390/cancers14071851)

The EGFR signaling modulates in mesenchymal stem cells the expression of miRNAs involved in the interaction with breast cancer cells

Supplementary material

Figure S1. Effects of miR-379-3p on the proliferation of breast cancer cell lines. The proliferation rates of MDA-MB-468 and MDA-MB-231 cells transfected with miR-379-3p mimic (A) or miR-379-3p inhibitor (B) and their respective non-targeting controls (NTCs) were assessed from 0 to 72 hours after transfection. Proliferation index was determined measuring OD at the indicated time points and calculating the ratio compared to zero time point (*P ≤0.05 and **P ≤0.005; Student's t-test).

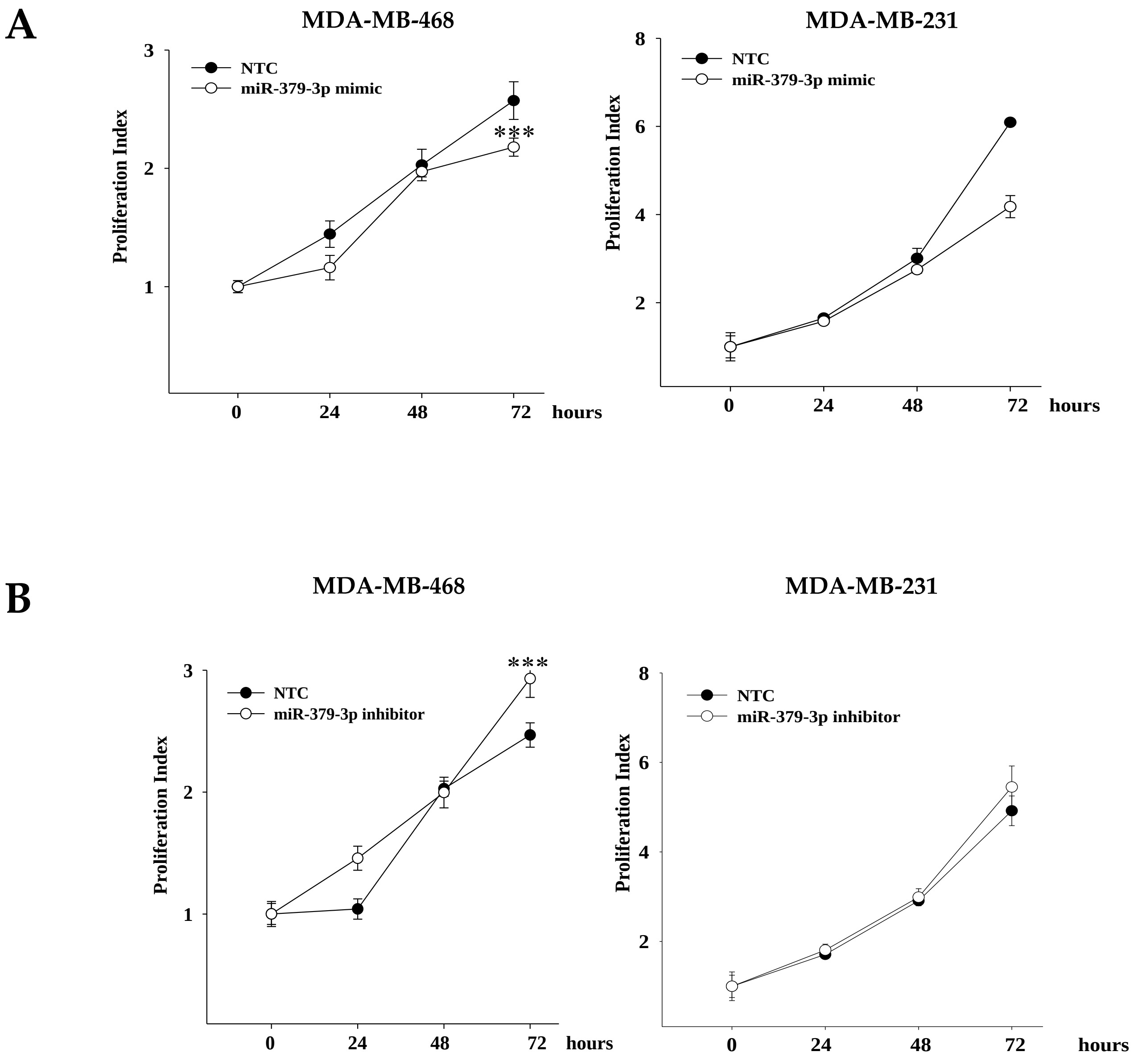

Figure S2. Effects of miR-379-3p on the ability of breast cancer cells to migrate and invade. MDA-MB-468 and MDA-MB-231 cells were transfected for 72 hours with miR-379-3p mimic (A) or inhibitor (B) and their respective non-targeting controls (NTCs) and then allowed to migrate through inserts toward serum containing medium for 16 hours (*P ≤0.05; Student's t-test). C) The invasive ability of MDA-MB-468 and MDA-MB-231 cell lines transfected miR-379-3p mimic or negative control (NTC) was determined using a Boyden chamber-based colorimetric assay (***P ≤0.001; Student's t-test).

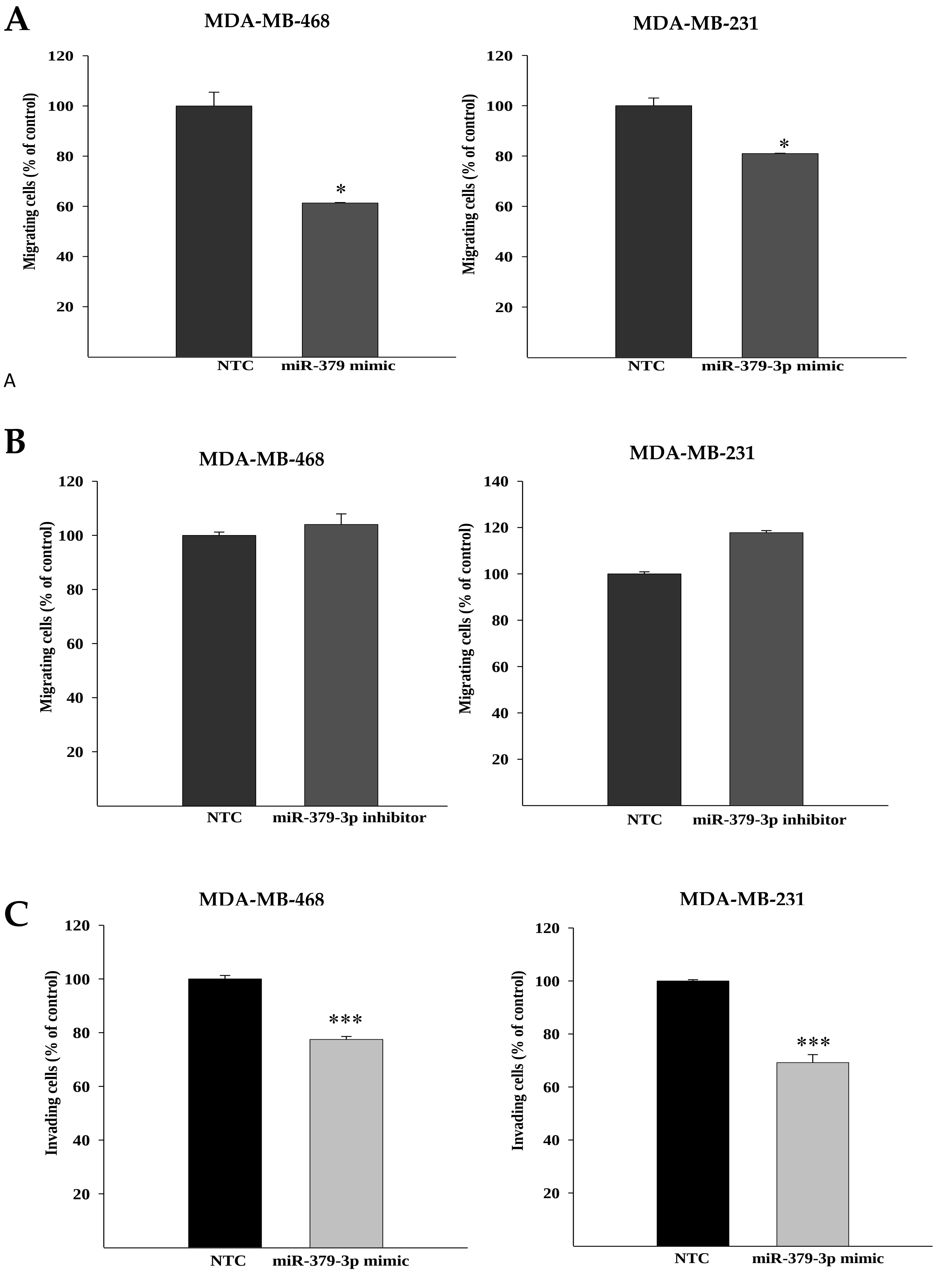

Figure S3. Original immunoblotting images. (A) Western Blot panels relative to figure 7B-C in the main text. (B) Western Blot panels relative to figure 7D-E in the main text. The original scans of the blots are shown. Squares indicate the images used for final figures. Densitometric analyses were reported in the figure 7 in the main text.

A

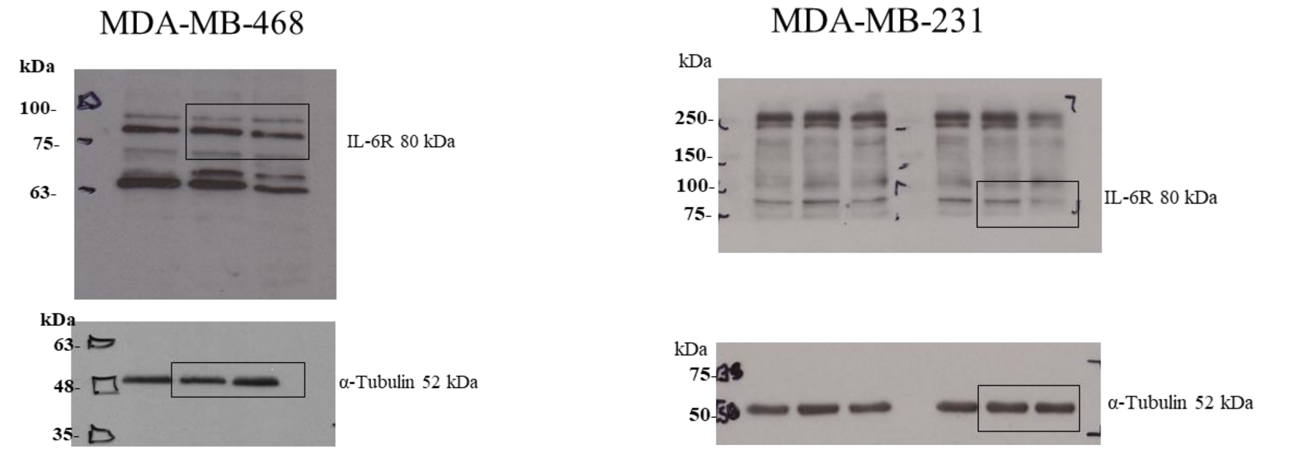

B

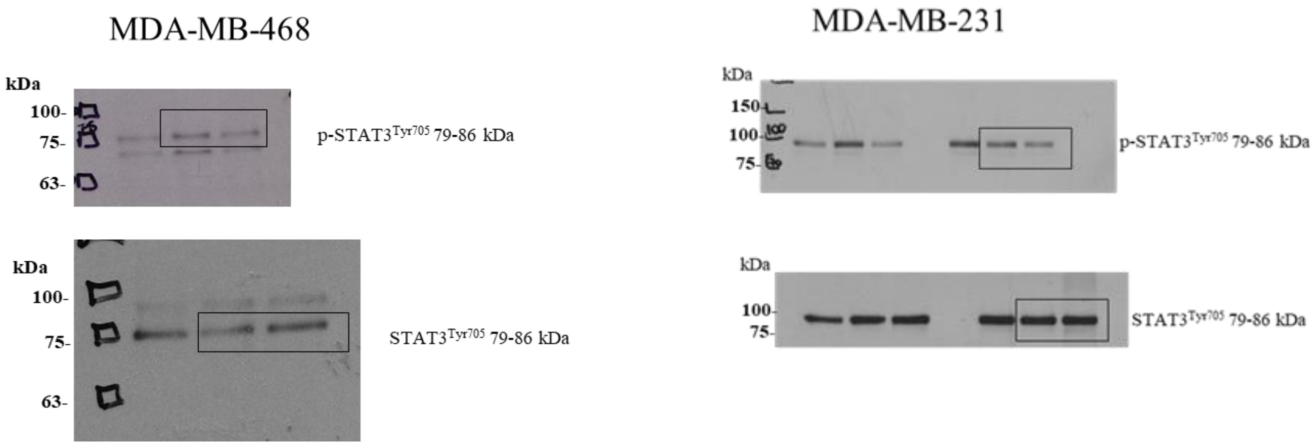

Supplement: Supplementary file 1 [file cancers-14-01851-s001.zip › Supplementary Figures.pdf]
